# Interventions on children’s and adolescents’ physical activity and sedentary behaviour: protocol for a systematic review from a sex/gender perspective

**DOI:** 10.1186/s13643-019-0963-2

**Published:** 2019-02-26

**Authors:** Yolanda Demetriou, Catherina Vondung, Jens Bucksch, Annegret Schlund, Carolin Schulze, Guido Knapp, Stephanie E. Coen, Lorri Puil, Susan P. Phillips, Anne K. Reimers

**Affiliations:** 1 Department of Sport and Health Sciences, Technical University of Munich, Georg-Brauchle-Ring 62, 80992 Munich, Germany; 2 Department of Natural and Human Sciences, Heidelberg University of Education, Heidelberg, Germany; 3 Institute of Human Movement Science and Health, Chemnitz University of Technology, Chemnitz, Germany; 4 Department of Statistics, Technical University Dortmund, Dortmund, Germany; 5 Department of Geography, Western University, London, Canada; 6 Department of Anesthesiology, Pharmacology & Therapeutics, Faculty of Medicine, University of British Columbia, Vancouver, Canada; 7 School of Medicine, Queen’s University, Kingston, Canada

**Keywords:** PRISMA-P, Girls, Boys, Randomized controlled trial, Youth, Cochrane, Exercise

## Abstract

**Background:**

Low levels of physical activity (PA) and high levels of sedentary behaviour (SB) have been observed in young people. Both behaviours are detrimental for health with patterns tending to continue into adulthood. There is sustained value in establishing health habits in early years. Even though levels of SB and participation in PA differ among boys and girls, and the effectiveness of interventions to promote PA and/or prevent sedentary behaviours varies by sex/gender to date, sex/gender in systematic reviews is not yet widely considered. Additionally, while tools have been proposed, there is no consensus on the criteria to assess sex/gender in systematic reviews in the context of health promotion. The main objectives of this systematic review are to evaluate the effects of interventions on girls’ and boys’ PA and SB and to appraise the extent to which the studies have taken sex/gender into account.

**Methods:**

Eleven electronic databases will be searched to identify all relevant (randomized) controlled trials. Two independent reviewers will screen studies, extract data and appraise the quality of studies. The main outcome of the studies will be a quantified measure of PA and/or SB. Risk of bias of individual studies will be assessed using the Cochrane Risk of Bias Tool for RCTs. Meta-analyses will be conducted when possible among studies with sufficient homogeneity. To evaluate sex/gender considerations in primary studies, we will use a sex/gender checklist that builds on existing tools and was developed during a 2-day, iterative process among a multidisciplinary panel of 16 experts. The GRADE framework will be used to evaluate evidence across studies for each main efficacy outcome.

**Discussion:**

To our knowledge, our systematic review will be the first to analyse how sex/gender is considered in interventions promoting PA and/or reducing SB in children and adolescents in detail. The review will provide information on how sex/gender aspects have been considered in recent research and the extent to which sex/gender might impact study outcomes. Our findings will be of interest to stakeholders, health promoters, researchers and policy makers who wish to support more equal outcomes from interventions promoting PA and/or reducing SB.

**Trial registration:**

PROSPERO CRD42018109528.

**Electronic supplementary material:**

The online version of this article (10.1186/s13643-019-0963-2) contains supplementary material, which is available to authorized users.

## Background

Globally, low levels of physical activity (PA) and a high degree of sedentary behaviour (SB) have been observed in young people [1–4]. These low levels of PA and high levels of SB are detrimental for health in this young age group [5, 6]. Additionally, patterns of PA and SB in childhood and adolescence tend to continue into adulthood during which there is strong evidence for health benefits of low SB and high PA [7, 8]. Therefore, it is vital to establish patterns of greater PA and less SB early in life.

In recent research, lower PA levels are consistently reported for girls compared to boys [1, 9–11]. These differences are meaningful across all age groups and in nearly all countries and regions. Interestingly, the difference in PA is greatest for vigorous PA, less marked for moderate PA and does not exist for light PA [12].

The picture for SB is different. International self-report data from 2002 and 2010 showed that electronic media use steadily increased across all countries surveyed in the Health Behaviour in School-aged Children (HBSC) study [12]. In 2010, boys reported about 6 h of sedentary screen time per day compared to about 5 h for girls. Girls more often use computers for social and academic purposes, whereas boys more frequently use them for gaming [10]. Objectively measured overall SB by the International Children’s Accelerometry Database (ICAD) showed that girls and boys sit up to 70% of their waking time, with slightly more SB for girls, but the difference between boys and girls consistently widens from childhood to adolescence [13].

Despite growing research and interventions to promote PA and decrease SB, sex/gender differences persist. This suggests that current interventions may not be sufficiently taking into account the evidence about sex/gender differences in PA and SB [10, 14]. A recent systematic review of the effectiveness of after-school programmes to enhance moderate-to-vigorous physical activity (MVPA) reported that a small minority of studies had conducted subgroup analyses in boys and girls with some evidence of greater effect on MVPA in boys. However, this finding was based on only the few studies that had compared sex/gender-specific findings [15]. Most systematic reviews of PA and SB interventions do not report on sex/gender. There is, therefore, a real need to evaluate this issue more rigorously.

With the introduction and expansion of PRISMA-E(quity), the field of health promotion is moving in the direction of becoming more sensitive to equity issues in systematic reviews and meta-analyses [16]. An equity framework also takes into account the fact that interventions can result in intervention-generated inequalities between different target groups (e.g. sex/gender groups), also referred to as the inverse law of evidence [17, 18]. Two scoping reviews related to sex/gender inequalities in adult PA interventions found some heterogeneities between the effects of interventions in men and women [19, 20]. Nevertheless, sex/gender is not yet widely considered in systematic reviews when appraising the existing evidence, potentially leading to suboptimal interventions for diverse populations [21].

Several theoretical approaches can be used to conceptualize gender and its relation to health [22–24]. The Canadian Institutes of Health Research define gender as “the socially constructed roles, behaviours, expressions and identities of girls, women, boys, men and gender diverse people” [25, 26]. Gender is considered multidimensional and dynamic and includes gender roles (behavioral norms), gender identities (how we see ourselves), gender relations (how we interact with each other) and institutionalized gender [26]. Although gender has been traditionally conceptualized as binary (feminine/masculine), there is growing recognition of the diversity with which individuals understand, experience and express gender on a fluid continuum [25]. Sex is a multidimensional biological construct that encompasses anatomy, physiology, genes and hormones [22]. Variation also exists in the biological attributes that comprise sex and how those attributes are expressed [25, 26]. The Cochrane Sex/Gender Methods Group, a subgroup of the Campbell and Cochrane Equity Methods Group, emphasizes that sex-based biological factors and gendered social factors influence each other and interactively shape health behaviour, opportunities and outcomes. In recognition of this theoretical and empirical entanglement, the group recommends using the term sex/gender [27, 28], terminology we have adopted in this protocol.

The main objectives of this systematic review are to evaluate the effects of interventions on girls’ and boys’ PA and SB, and to appraise the extent to which the studies have taken sex/gender into account. To reach this aim, all primary studies included in the review will be assessed based on the previously mentioned sex/gender checklist developed by the authors of this manuscript in cooperation with an international expert group (please contact the corresponding author for the current version of the checklist).

## Methods

This systematic review protocol is registered in the PROSPERO international prospective register of systematic reviews (registration number: CRD42018109528). We prepared the protocol using the Preferred Reporting Items for Systematic Reviews and Meta-Analysis Protocols (PRISMA-P) 2015 statement [29] (Additional file 1). The final review will be reported using the Preferred Reporting Items for Systematic Reviews and Meta-Analysis (PRISMA) statement and its equity extension (PRISMA-E) as guidance [16]. Important protocol amendments will be documented and published with the results of the review.

### Search strategy

The search strategy will be based on Cochrane standards and designed in collaboration with a Cochrane information specialist [16, 30]. The Cochrane Central Register of Controlled Trials (CENTRAL) via Ovid; Ovid MEDLINE, Epub Ahead of Print, In-Process and other Non-Indexed Citations, Daily, and Versions; Ovid Embase; Science Citation Index Expanded (SCI-EXPANDED); Clarivate Web of Science; Conference Proceedings Citation Index (CPCI-S); EBSCO PsycINFO, EBSCO Eric, EBSCO SPORTDiscus; and ProQuest Dissertations & Theses Global will be searched. The subject strategies for databases will be based on the MEDLINE search strategy (Appendix), which will combine Medical Subject Heading (MeSH) terms and keywords related to physical activity and sedentary behaviour. We will also search ClinicalTrials.gov and the World Health Organization (WHO) International Clinical Trials Registry Platform (ICTRP) to identify ongoing or recently completed studies.

Reference lists of existing systematic reviews, identified by searching Epistemonikos, will be cross-checked to ensure all studies are identified. Experts in the field will also be contacted to identify potentially eligible studies. The included primary studies will be complemented by additional information from, e.g. study protocols and/ or materials to identify all relevant aspects of the intervention components.

### Eligibility criteria

We will include randomized controlled trials (parallel group or cluster-randomized) and controlled trials in the systematic review. The main outcomes will be quantified PA and/or SB by any type of measure (e.g. self-reported, accelerometer data) in children and adolescents within the average age range of 3–19 years. Studies only targeting children and adolescents with specific health issues will be excluded. Additionally, we will exclude college and university students because this population group represents the beginning of a new life stage. The aim of the intervention programmes must be promotion of informal and formal PA behaviours and/or the reduction of SB in children and adolescents. Additionally, all intervention studies must have reported sex/gender disaggregated PA and/or SB at baseline and/or follow-up, and/or explained how they dealt with sex/gender during the outcome analysis (e.g. sex/gender adjusted analysis), and/or reported that there were no differences in the outcome when looking at sex/gender. The comparators should either be an active control group for example receiving an intervention to promote children’s creativity or cognitive performance without components promoting PA or reducing SB or a control group with no intervention. In order to base the results of the systematic review on current activities, only studies published after the year 2000 will be included. Due to resources and time constraints, we will restrict eligibility to published peer-reviewed studies in the English language.

### Study selection

Two independent reviewers will screen all identified references for inclusion against eligibility criteria. All records will be imported into Covidence, and duplicates will be removed automatically from the software. In the first step of screening, titles and abstracts will be screened to remove clearly irrelevant records. In the second step, the full text of citations that are considered of potential or uncertain relevance by the two reviewers will be retrieved. Any disagreements during the study selection process will be resolved by a third independent reviewer or if necessary by discussions of the three reviewers after re-examination of the articles. If full texts are not available or additional data are needed to determine eligibility, authors will be contacted via e-mail. A maximum of two contact attempts will be made. Additionally, after searches and study selection are conducted, we will contact experts in the field to determine further studies that meet the inclusion criteria.

### Data extraction

All data extraction will be carried out by two independent reviewers and any discrepancies resolved through discussion or adjudication by a third reviewer if consensus is not reached. To ensure consistency of data extraction across reviewers, we will pilot a data extraction spreadsheet. For each study, specific details will be extracted. First, information about general study characteristics, description of study sample and dropout rate, intervention content details and intervention approaches will be extracted. Second, we will extract intervention outcomes, measurement points and instruments as well as sample size calculation and confounders taken into account to analyse the effectiveness of the intervention on PA and/or SB outcomes. Reviewers will not be blinded to authors or journals when extracting the data. If information is missing or clarification of data is required, authors will be contacted via e-mail. A maximum of two contact attempts will be made. The process in which sex/gender will be appraised and transferred to data extraction is described below (see “sex/gender checklist”).

### Quality assessment and risk of bias

For the assessment of the risk of bias of the primary studies, we will use the Cochrane Risk of Bias Tool for RCTs [30, 31] for both RCTs and controlled clinical trials. The tool is a domain-based evaluation, in which critical assessments will be made separately for sequence generation (selection bias), allocation sequence concealment (selection bias), blinding of participants and personnel (performance bias), blinding of outcome assessment (detection bias), incomplete outcome data (attrition bias), selective outcome reporting (reporting bias) and other potential sources of bias. The judgment for each entry will involve assessing the risk of bias as “low risk”, “high risk” or “unclear risk”, with the last category indicating either lack of information or uncertainty about the potential for bias. Controlled clinical trials will be considered to be at high risk of bias for domains related to randomization. Quality assessment will be done by two independent reviewers, and discrepancies resolved through discussion or adjudication by a third reviewer if consensus cannot be reached.

### Sex/gender checklist

To assess the degree to which sex/gender was considered in intervention studies that promote PA and/or reduce SB in participants, we developed a comprehensive sex/gender checklist in a three-step procedure. First, the existing literature [22, 32–35] and tools [36–40] that appraise sex/gender in research were collated, including existing guidance for systematic review authors [41, 42]. Second, we summarized existing instruments and checked them for applicability to our objectives. Third, the first draft of the sex/gender checklist was set up and finalized in collaboration with international experts in the field of sex/gender sciences and methodology (e.g. members of the Cochrane Sex/Gender Methods Group, a subgroup of the Campbell and Cochrane Equity Methods Group). The current version of the sex/gender checklist consists of 16 items in the following categories: background and concepts, study design, intervention planning and delivery, statistical accounting and presentation and interpretation of findings. These items are rated using three categories by item-specific definitions and provide information on the extent to which the primary study took sex/gender into account regarding the respective item.

### Statistical analyses

Meta-analyses for interventions promoting PA and/or reducing SB will be undertaken if the studies are sufficiently similar clinically and methodologically, otherwise a semi-quantitative or narrative synthesis will be conducted. Possible estimated effect sizes for the controlled intervention studies may be, e.g. Cohen’s *d* for the standardized mean difference of PA or SB before and after the intervention, separately reported for boys and girls or interaction effects for boys and girls from regression models. For all reported estimated effect sizes, a measure of precision like standard error or confidence interval must be available. The inclusion of cluster-randomized trials in the meta-analyses will be handled appropriately [43]. The random-effects meta-analysis model will be the first model considered for combining the estimated effect sizes. The assumption of a common effect size in all the studies is too restrictive due to different designs of intervention studies or of the interventions used, or the frequency or duration of the intervention. If substantial heterogeneity between the studies is present, moderator variables will be examined in the context of meta-regression. As there is the danger of overfitting in meta-regression, at least ten relevant studies should be available for a meta-regression with one moderator variable. Meta-regression techniques will also be used to judge the effect of high risk of bias.

If the meta-analyses produce significant results, publication bias will be examined. First, we will address the possible effect of publication bias using a fail-safe number, e.g. the number of non-published studies that would reverse the significant meta-analysis result into a non-significant one. If this fail-safe number is reasonable, that is, not too large, the Copas selection model may be used for a sensitivity analysis along with the Henmi-Copas confidence interval approach [44].

All meta-analysis models will be analysed in the freely available statistical software R using various packages (e.g. meta, metafor, metasens, metaplus, CAMAN).

The quality of evidence across studies will be assessed for each outcome as high, moderate, low or very low using the GRADE framework [45]. With the GRADE approach, RCT evidence starts at the highest quality level but may be downgraded based on an assessment of the following domains: study limitations (risk of bias), imprecision, heterogeneity, indirectness and suspicion of publication bias. Controlled clinical trials that are not randomized will be downgraded based on risk of bias due to lack of randomization and start at moderate quality of evidence. GRADEpro|GDT software will be used to create a summary of findings table and rate the quality of the evidence using the GRADE framework.

## Discussion

To our knowledge our systematic review will be the first to systematically assess how sex/gender is considered in interventions promoting PA and/or reducing SB in children and adolescents. The review will provide information on how sex/gender has been considered or reported in recent research and to what extent these might have an impact on the study outcomes.

We anticipate this systematic review will lead to several publications based on a socio-ecological perspective and according to the U.S. Guide to Community Preventive Services in order to provide a better understanding regarding the influence of different types of intervention programmes [46]. Additionally, a briefing note on appraising interventions that consider sex/gender in promoting PA or reducing SB will be published. This briefing note will be jointly developed with the Cochrane Sex/Gender Methods Group and distributed to the Cochrane Collaboration via the Campbell and Cochrane Equity Methods Group.

Our findings will be of interest to stakeholders and health promoters as well as researchers and policy makers who wish to foster gender equity in interventions promoting PA and/or reducing SB. Therefore, a workshop will take place where we will convert our primary scientifically oriented sex/gender checklist and the results of this systematic review into practical documents that aid others in appropriately integrating sex/gender considerations in interventions.

The results of the review will help establish sex/gender guidelines on the development, implementation and appraisal of PA promotion and SB reduction interventions. The project will build both the field of PA promotion and SB prevention and methodology for conducting systematic reviews using a sex/gender lens. The results will be disseminated to academic and non-academic audiences through peer-reviewed publications, conferences and formal presentations and in formal meetings.

### Additional file

Additional file 1: Preferred Reporting Items for Systematic Review and Meta-Analysis Protocols (PRISMA-P) 2015 checklist: recommended items to include in a systematic review protocol. (DOC 84 kb)

## Appendix

**Table 1:** Sample MEDLINE search strategy

Number	Search terms
1	Adolescent/ (1879830)
2	exp Child/ (1783242)
3	(adolesc$ or boy? or child$ or girl? or juvenile? or kid? or school$ or school age$ or student? or teen$).ti,kf. (1090760)
4	(adolesc$ or boy? or child$ or girl? or juvenile? or kid? or school$ or school age$ or student? or teen$ or youth?).ab. /freq = 2 (908853)
5	or/1-4 (3245722)
6	exp Motor Activity/ (259691)
7	exp Exercise/ (168655)
8	exp Exercise Therapy/ (43456)
9	exp Recreation/ (187698)
10	exp *Sports/ (113371)
11	Physical Exertion/ (55439)
12	exp Physical Fitness/ (26257)
13	exp “Play and Playthings”/ (12587)
14	(active or activities or activity or aerobic? or athletic? or badminton or baseball or basketball or bicycl$ or bike? or biking or boxing or cardio or cricket or cycling or dance or dancing or exercis$ or fitness or football or gymnastic? or handball or hockey or jogging or jiu jitsu or judo or jujitsu or karate or playground? or rugby or running or soccer or sport? or swim$ or tennis or training or volleyball or walk? or walking or yoga).tw,kf. (4104009)
15	physical$ activ$.tw,kf. (97509)
16	Sedentary Lifestyle/ (6980)
17	Television/ (12915)
18	(gaming or television or tv or video game? or videogame?).tw,kf. (27953)
19	or/6-18 (4250380)
20	intervention?.ti. and 19 (26682)
21	((amount? or effect? or effectiveness or encourag$ or evaluat$ or impact? or improve$ or improving or increase? or increasing or intervention? or modif$ or promot$) adj3 (activity level? or exercise or fitness or mobility or physical activit$ or step$)).ti,kf. (24388)
22	((amount? or effect? or effectiveness or encourag$ or evaluat$ or impact? or improve$ or improving or increase? or increasing or modif$ or promot$) adj4 (activity level? or exercise or fitness or mobility or physical activit$ or step$)).ab. (131764)
23	((avoid$ or curb$ or decreas$ or discourag$ or effect? or effectiveness or eliminat$ or evaluat$ or impact? or modif$ or prevent$ or reduc$) adj3 (computer$ or inactiv$ or screen-based or screen time or sedentary or sitting or television or tv or video game?)).ti,kf. (4977)
24	((avoid$ or curb$ or decreas$ or discourag$ or effect? or effectiveness or eliminat$ or evaluat$ or impact? or modif$ or prevent$ or reduc$) adj4 (computer$ or inactiv$ or screen-based or screen time or sedentary or sitting or television or tv or video game?)).ab. (31431)
25	or/20-24 (196821)
26	5 and 25 (30682)
27	randomized controlled trial.pt. (466685)
28	controlled clinical trial.pt. (92572)
29	randomi?ed.ab. (501162)
30	placebo.ab. (190972)
31	clinical trials as topic/ (184490)
32	randomly.ab. (295330)
33	trial.ti. (186030)
34	(allocation or allocated).ab. (91828)
35	assigned.ab. (212727)
36	(controlled adj2 (study or trial)).ab. (130228)
37	control group?.ab. (399548)
38	((singl$ or doubl$) adj blind$).ab. (141426)
39	or/27-38 (1625780)
40	animals/ not (humans/ and animals/) (4453468)
41	39 not 40 (1431749)
42	26 and 41 (8753)
43	limit 42 to yr = “2000 - 2018” (7727)

## Abbreviations

MVPA: Moderate-to-vigorous physical activity
PA: Physical activity
PRISMA: Preferred Reporting Items for Systematic Reviews and Meta-Analysis
PRISMA-E: Preferred reporting items for systematic review and meta-analysis Equity
PRISMA-P: Preferred reporting items for systematic review and meta-analysis protocols
SB: Sedentary behaviour
WHO: World Health Organization
